# Heme-Oxygenase 1 Mediated Activation of Cyp3A11 Protects Against Non-Steroidal Pain Analgesics Induced Acute Liver Damage in Sickle Cell Disease Mice

**DOI:** 10.3390/cells14030194

**Published:** 2025-01-28

**Authors:** Ravi Vats, Ramakrishna Ungalara, Rikesh K. Dubey, Prithu Sundd, Tirthadipa Pradhan-Sundd

**Affiliations:** 1 Versiti Blood Research Institute and Blood Center of Wisconsin, 8733 West Watertown Plank Road, Milwaukee, WI 53226, USA; ravi.vats77@gmail.com (R.V.); veu5@pitt.edu (R.U.); rdubey@versiti.org (R.K.D.); psundd@versiti.org (P.S.); 2 Divisions of Cell Biology, Neurobiology and Anatomy and Bioengineering, Medical College of Wisconsin, Milwaukee, WI 53226, USA

**Keywords:** sickle cell disease, non-steroidal analgesics, pain management, heme oxygenase 1, Cyp3A11, Kupffer cells

## Abstract

Pain constitutes a significant comorbidity associated with sickle cell disease (SCD). Analgesics serve as the primary method for pain management; however, the long-term effects of these drugs on the liver of SCD patients remain not completely understood. Using real-time intravital imaging, we analyzed the effect of non-steroidal analgesics (NSA) in the liver of control and SS (SCD) mice. Remarkably, we found completely opposing effects in the liver of control and SS mice post-NSA treatment. Whereas SS mice were able to better tolerate the NSA treatment acutely compared to their littermate controls, in the long term, these mice showed delayed resolution of liver injury and exacerbated fibrosis compared to control mice. Mechanistically, we found that SS mice were protected from cytotoxicity caused by NSA at baseline due to the significant activation of hepatic Kupffer cells, which produced heme-oxygenase 1 (HO-1). HO-1 promoted the activation of the cytoprotective enzyme Cyp3A11, which inhibited hepatic damage caused by NSA. However, in the long term, depletion of hepatic Kupffer cells led to reduced expression of HO-1, which blocked the activation of Cyp3A11, resulting in fibrosis and a delay in the resolution of liver injury and inflammation. These preclinical data provide a strong proof-of-concept for HO-1 as well as Cyp3A11 as cytoprotectors against NSA-induced liver damage in the Townes model of SCD and support further development of these compounds as potential novel therapies for end-organ damage in SCD.

## 1. Introduction

Pain is the hallmark of sickle cell disease (SCD) and the most common reason for hospital visits [1,2]. Individuals with SCD experience both acute and chronic pain throughout their lifetime [1,2,3,4]. Acute pain arises from repeated episodes of vaso-occlusive crises (VOC), while the etiology of chronic pain is not completely understood [5]. Analgesics (opioids as well as non-opioids) serve as the primary method for pain management [6,7,8]; however, the long-term effect of these drugs in the liver and kidney of SCD patients is not completely understood. Hydroxyurea is the current FDA-approved drug to mitigate or decrease the pain crisis in SCD patients [9,10,11]. Recently, there have been major advances in understanding the potential usefulness of analgesics as well as natural compounds in mitigating SCD-induced pain crises [12,13,14]. Although analgesics are frequently used as a palliative therapy to deal with SCD-induced pain crises, the short-term and long-term effects of these drugs in promoting organ damage (liver and kidney) in SCD and the body’s adaptive mechanism to mitigate the harmful effects of these drug metabolites are not completely understood.

Heme oxygenases (HO) are inducible, rate-limiting enzymes with three different isoforms solely responsible for heme degradation [15,16,17]. Heme oxygenase 1 (HO-1) can be stimulated by a diverse group of stimuli (including oxidative stress, cytokines, heavy metals, hypoxia, nitric oxide, and heme) compared to the other two isoforms [18,19,20]. SCD patients were shown to have higher expression of HO-1 in the renal tubules of the kidney and the liver [21,22,23]. Along with heme degradation, HO-1 has several other cytoprotective roles in the body, including anti-oxidation, vascular tone modification, anti-inflammatory function, and downregulation of adhesive molecules expressed in the liver and spleen [24,25,26]. Several preclinical research have established the role of HO-1 in reducing SCD severity [27,28,29]. However, its hepatoprotective role against analgesic-induced liver damage in SCD/SS mice is not completely known.

Using real-time intravital imaging, we investigated the effect of non-steroidal analgesics (NSA) in the liver of control (AS) and sickle (SS) mice. Remarkably, we found opposite effects in the liver of control and SS mice post-NSA administration. Whereas SS mice were able to better tolerate the NSA treatment acutely compared to their littermate controls, in the long term, these mice showed delayed resolution of liver injury and exacerbated fibrosis compared to control mice. Mechanistically, we found that SS mice were protected from cytotoxicity caused by NSA at baseline due to significant activation of hepatic Kupffer cells, which produced HO-1. HO-1 promoted the activation of cytoprotective enzyme Cyp3A11 in a nuclear factor erythroid 2-related factor 2 (NRF2)-independent manner, which accelerated the degradation of NSAs. However, a persistent reduction of hepatic Kupffer cells resulted in decreased production of HO-1, which blocked Cyp3A11 activation in the liver, increasing fibrosis and delaying the resolution of liver injury and inflammation in the long run. These preclinical data provide a proof-of-concept for HO-1 as well as Cyp3A11 as cytoprotectors against non-steroidal pain analgesic-induced liver damage in the Townes model of SCD and support further development of these compounds as potential novel therapies for end-organ damage in SCD.

## 2. Methods

Animals and treatment: Townes SS mice (SS, homozygous for Hba^tm1(HBA)Tow^, homozygous for Hbb^tm2(HBG1,HBB*)Tow^) and non-sickle control mice (AS, homozygous for Hba^tm1(HBA)Tow^, compound heterozygous for Hbb^tm2(HBG1,HBB*)Tow^/Hbb^tm3(HBG1,HBB)Tow^) [21] were obtained from the Jackson Laboratory (Bar Harbor, ME, USA) and housed in a specific pathogen-free animal facility. The Institutional Animal Care and Use Committee at the Medical College of Wisconsin and the University of Pittsburgh had approved all animal experiments. An equal number of males and females were always used in these experiments, and unless otherwise stated, all the mice used were 3 months old.

NSA administration: The AS and SS mice were fasted for 24 h without food and bedding but with free access to water. The mice were intraperitoneally injected with 350 mg/kg body weight NSA (acetaminophen (obtained from Sigma, Carlsbad, CA, USA)) and had free access to food and water after the injection. Liver tissues and blood were collected 6 h, 24 h, 48 h, 72 h, 7 days, and 14 days post-NSA injection, and the deaths of the animals were recorded every 6 h over a 72 h period. ALT and AST were measured using commercially available kits from Abcam (Waltham, MA, USA).

Oxyhemoglobin treatment: OxyHb (10 μm/kg) was administered intravenously (IV injection) via the tail vein. Mice were sacrificed after 6 h, and liver and blood samples were collected for further study.

LPS treatment: A dose of 0.1 μg/kg LPS was administered intravenously in AS and SS mice. Mice were sacrificed after 3 h, followed by liver and blood samples collection for further analysis.

Clodronate treatment: Clodronate liposome (100–150 μl/mice) was injected into AS and SS mice via IP route. Mice were sacrificed within 24–48 h of injection, and liver and blood samples were collected for further analysis.

Haptoglobin administration: 20 μmole/kg haptoglobin (Sigma Aldrich; Cat# SRP6507-1MG) was injected into AS and SS mice intravenously. Mice were sacrificed after 3 h, and liver and blood samples were collected for further analysis.

Iron dextran treatment. AS and SS mice were administered 10 doses (1 dose/alternate day) of 100 mg/kg body weight iron dextran (Sigma D8517-25ML) intraperitoneally. Mice were euthanized after 3 weeks, and liver and blood samples were collected for further characterization.

Surgical preparation and quantitative liver intravital imaging (qLIM): The surgical method was applied according to the method previously published by R. Vats et al. [30,31,32]. Intravascular fluorescent dyes included 200 μg of Texas red (TXR) dextran (sigma), which was used to visualize the blood flow through the hepatic sinusoids, and AF-F4/80 Cy3 was used for marking the hepatic Kupffer cells. Microscopy was performed using a Nikon MPE multi-photon excitation microscope.

Co-immunoprecipitation assay: Co-immunoprecipitation was performed using the Pierce^®^ Crosslink Immunoprecipitation Kit (cat # 26147, ThermoFisher Scientific, Waltham, MA, USA), following the manufacturer’s instructions. Briefly, anti-HO-1 and normal mouse IgG were each cross-linked to Pierce Protein A/G Plus Agarose by incubation at room temperature for 1 h on an end-to-end rotor. The mixtures were centrifuged in a spin column to fix the antibody–resin complexes. After sequential washes with quenching buffer, sodium cyanoborohydride solution, 1× coupling buffer, and wash solution, the immobilized antibodies were prepared for co-immunoprecipitation. To prevent co-elution of the antibody with the antigen, the coupled antibody was incubated with DSS (disuccinimidyl suberate) for 30 min. Liver lysates were precleared with Control Agarose Resin by incubation at 4 °C for 1 h. For immunoprecipitation, the antibody-cross-linked resin was washed with IP wash/lysis buffer, and the precleared liver lysate was added. The mixture was incubated overnight at 4 °C with gentle end-over-end mixing. After washing with wash buffer, the antigen was eluted using elution buffer and analyzed by immunoblotting.

Western blot: The liver protein lysate was separated by 4–12% Bis-tris gel and transferred onto a nitrocellulose membrane. Following blocking with 5% skim milk, the blots were probed with various primary antibodies and then probed with horseradish peroxidase (HRP)-conjugated secondary antibodies. The immunoreactive bands were visualized with enhanced chemiluminescence detection reagent using an Amersham imager (GE Healthcare, Chicago, IL, USA). GAPDH served as an internal control for total protein. The intensity of the bands was quantified using ImageJ software v5. The antibodies used in western blot analysis are listed in Supplementary Table S1.

Immunohistochemistry and imaging: Frozen liver sections from control and SS mouse livers were used for immunohistochemistry. Briefly, frozen sections were fixed using paraformaldehyde followed by blocking in BSA for 45 min. Slides were then treated with primary and secondary antibodies. Slides were mounted in mounting media containing DAPI. Slides were imaged using NIKON confocal microscopy. The antibodies used in IHC analysis are listed in Supplemental Table S2.

mRNA isolation and qRT-PCR: mRNA was isolated and purified from the livers of AS and SS mice at the baseline and post-NSA treatment (n = 4–5/group). mRNA was isolated using Trizol (Invitrogen, Waltham, MA, USA). qRT-PCR was performed as described elsewhere [33]. 18S and GAPDH were used to normalize the mRNA expression data. The sequences of the primers are shown in Supplemental Table S3.

Statistical analysis: All comparisons between two groups were considered statistically significant by performing unpaired two-tailed Student’s *t*-test if *p* < 0.05 (*) or *p* < 0.01 (**). All calculations were performed with Prism version 7.0a (GraphPad Software). Error bars represent the standard deviation.

## 3. Results

### 3.1. Real-Time Intravital Imaging Reveals Significantly Less Hepatovascular Damage in SS Mice than Control Mice Post-Acute Overdose of NSA

NSA-induced acute liver injury is divided into two phases: (i) the injury phase and (ii) the resolution phase [34,35]. Previous studies have shown that 6–48 h post-NSA injection, the overall injury, mortality, and liver fibrosis are remarkably high in murine models, which starts to resolve around 72 h time point [36]. By 7 days post-NSA treatment, most of the hepatocytes undergo regeneration, and liver injury is completely resolved. By the 14-day time point, the liver cells return to normal, and there is no sign of injury or inflammation.

**Figure 1:**
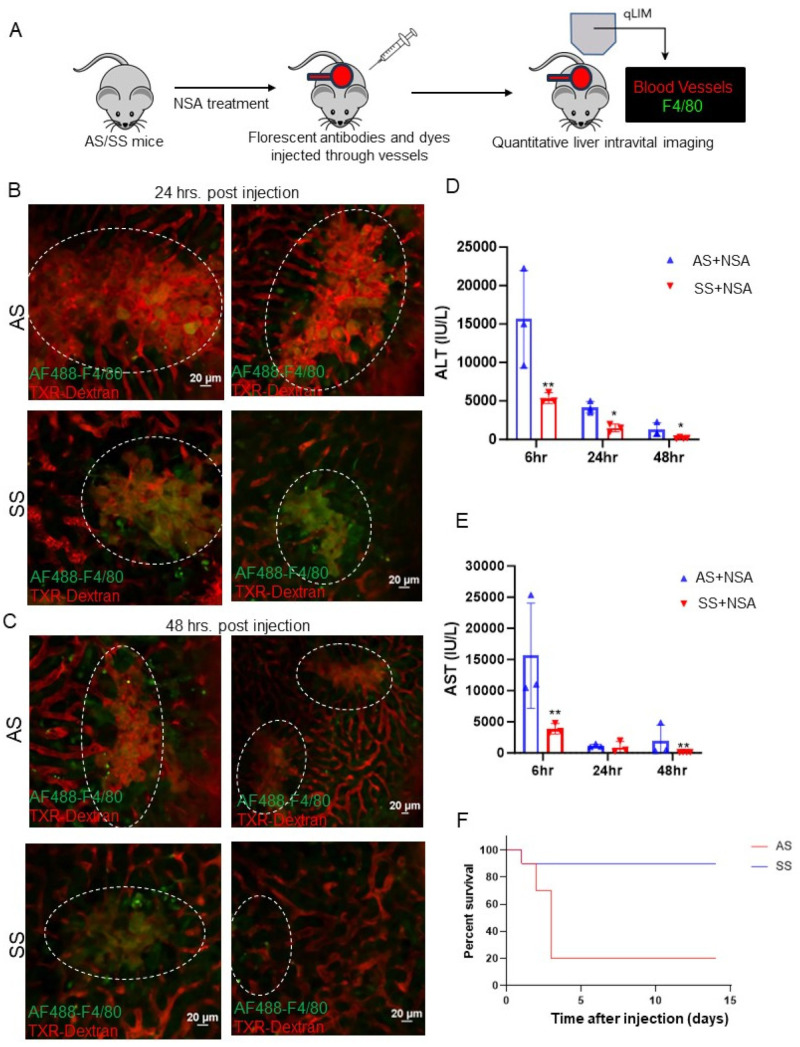
Non-steroidal analgesic (NSA)-associated liver injury progression is less severe in SS mice compared to littermate control mice. (**A**) Schematic of real-time intravital imaging to examine blood flow and Kupffer cell localization using Texas red dextran and F4/80, respectively. (**B**) Real-time intravital imaging shows vascular damage and blood flow stasis in control mouse liver, which appeared less severe in SS mouse liver at 24 h post-NSA administration. (**C**) Real-time intravital imaging exhibits persistently exacerbated vascular damage and blood flow stasis in control mouse liver, which appeared less severe in SS mouse liver even at 48 h post-NSA administration. (**D**) Bar graph depicting quantification of serum markers of liver injury (alanine aminotransferase; ALT) post-NSA administration at 6 h, 24 h, and 48 h. Remarkably, control mice show significant upregulation of ALT compared to SS mice at all given time points. (**E**) Bar graph depicting quantification of serum markers of liver injury (aspartate aminotransferasel; AST) post-NSA administration at 6 h, 24 h, and 48 h. Control mice show significant upregulation of AST compared to SS mice at all given time points. (**F**) Kaplan–Meir survival graph showing significant mortality of control mice post-NSA treatment, which was not seen in SS mice. *p*-value of significance * < 0.5. ** < 0.1. N = 3 mice. Statistical test performed: Student’s *t*-test. Scale bar: 0.2 μm.

All prior preclinical studies using the NSA model have described the biochemical changes in the liver. However, the extent of vascular injury and damage has not been previously described. Using real-time intravital imaging of the liver of live control and SS mice, we examined the extent of hepatovascular damage post-NSA administration. Texas red dextran (TXR dextran) was used to view the blood flow through hepatic vessels, and AF-F4/80-Cy3 was used to visualize the localization of hepatic Kupffer cells. The schematic of the experiment is shown in Figure 1A. Remarkably, 24 h post-NSA treatment, there was a significant stasis of blood flow in control mice associated with vascular damage and distortion of hepatic vessels (Figure 1B, Supplemental Movies S1 and S2). Although SS mice also showed some degree of vascular damage, the overall area of damage was significantly less in SS mouse liver, as shown in Figure 1B (Supplemental Movies S3 and S4). This was further confirmed using immunohistochemistry for hematoxylin and eosin (H&E) and Sirius red (S Red) staining, which showed significantly more liver damage in control mouse liver compared to SS mouse liver at 6 h and 24 h post-NSA administration (Figure S1A,B).

Similarly, at 48 h post-NSA administration, control mice exhibited exacerbated hepatovascular damage, complete loss of blood flow, and large areas of necrosis compared to SS mouse liver (Figure 1C). H&E and Sirius red staining further confirmed aggravated liver damage in control mice compared to SS mice (Figure S2A). Serum markers of liver injury including alanine aminotransferase (ALT) and aspartate aminotransferase (AST) showed significantly elevated levels in control mice at 6 h, 24 h, and 48 h time points compared to SS mice post-NSA treatment (Figure 1D,E). Finally, as reported by others [37], we found significantly high mortality in control mice up to 6 h post-injection, which was not seen in SS mice (Figure 1F). Taken together, these data show that SS mice can better tolerate NSA overdose and exhibit less hepatovascular damage and necrosis compared to control mice.

### 3.2. Real-Time Intravital Imaging Reveals Delayed Long-Term Injury Resolution in SS Mice Compared to Control Mice

**Figure 2:**
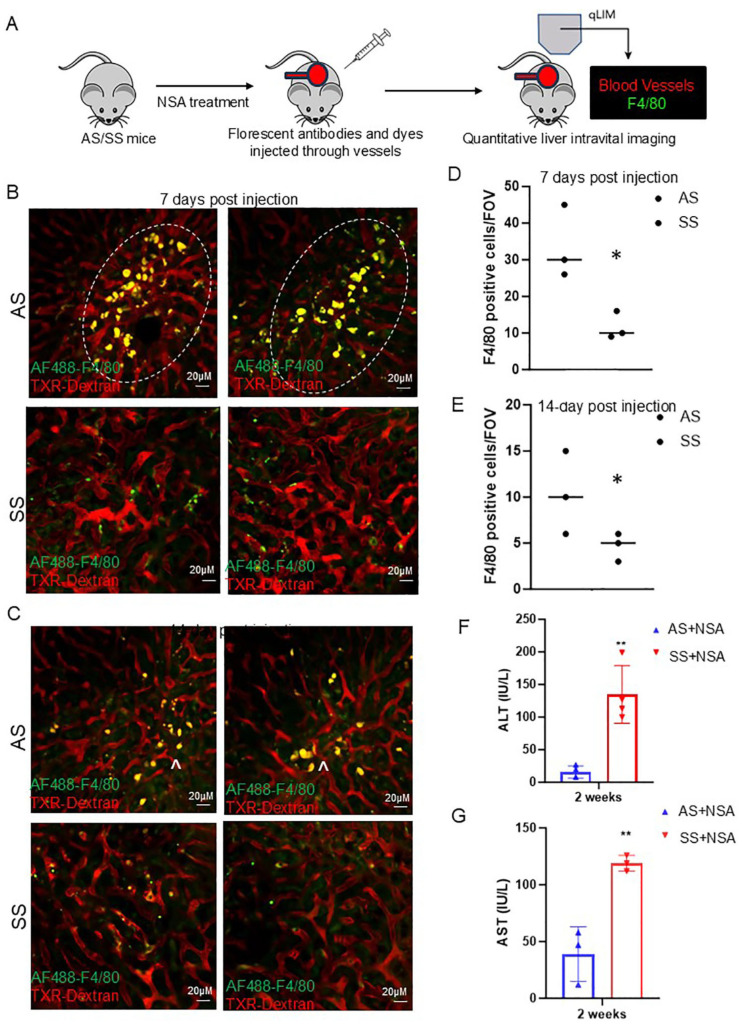
Resolution of non-steroidal analgesic (NSA) associated liver damage is delayed in SS mice. (**A**) Schematic of real-time intravital imaging to examine blood flow and Kupffer cell localization using Texas red dextran and F4/80, respectively. (**B**) Real-time intravital imaging exhibits a significant amount of Kupffer cell activation (indicated by ^) in control mice at 7 days post-NSA administration, which was associated with less vascular damage. Whereas in SS mice, Kupffer cell activation was not seen along with impaired hepatic blood flow. (**C**) Real-time intravital imaging showing persistent Kupffer cell activation in control mice at 14 days post-NSA administration and resolution of vascular damage and blood flow. Whereas in SS mice, Kupffer cell activation was not seen along with impaired blood flow. (**D**,**E**) Bar graph depicting quantification of Kupffer cell number in control and SS mice liver at 7- and 14-day post-NSA administration. (**F**,**G**) Bar graph depicting quantification of serum markers of liver injury alanine aminotransferase (ALT) and aspartate aminotransferase (AST) post 14 days of NSA administration. Remarkably, control mice show significant amelioration of AST and ALT compared to SS mice at the 14-day time point. *p*-value of significance * < 0.5. ** < 0.1. N = 3 mice. Statistical test performed: Student’s *t*-test. Scale bar: 0.2 um.

As we see protection against NSA-induced acute hepatovascular damage in SS mice, we next examined the long-term injury resolution caused by NSA in control and SS mouse liver (Figure 2A). Previous studies have established that by day 7, around 80% of all injury caused by NSA is reversed, whereas by day 14, the liver appears completely normal [34]. As shown in Figure 2B, the blood flow and hepatic vasculature appeared significantly normal in control mice at day 7 compared to 24 or 48 h time points (Figure 1B,C). Remarkably, along with amelioration of blood flow and hepatovascular injury, we also found a significant enhancement of hepatic Kupffer cells in control mice at day 7 and 14 post-NSA treatment (Figure 2B,C, Supplemental Movies S5–S12). In contrast, SS mice still showed impaired hepatic blood flow, which was associated with the reduction of hepatic Kupffer cell number. Quantification of the Kupffer cell marker C-type lectin receptor CLEC4F further confirmed a significant reduction in SS mice at both 7- and 14-day time points compared to AS mice (Figure 2D,E). When examined, serum markers of liver injury (AST and ALT) showed significant upregulation in SS mice, which were reduced and within the normal range in control mice (Figure 2F,G). Control mouse liver at this time point appeared normal by immunohistochemistry (IHC) staining of H&E and Sirius red, as shown in Figure S3A,B. Altogether, our data suggests that long-term resolution of NSA-induced liver damage is delayed in SS mice compared to control mice due to the absence of hepatic Kupffer cell number as well as Kupffer cell activation.

### 3.3. Hepatic Kupffer Cell Activation Protects Against NSA-Induced Hepatic Damage in SS Mice

**Figure 3:**
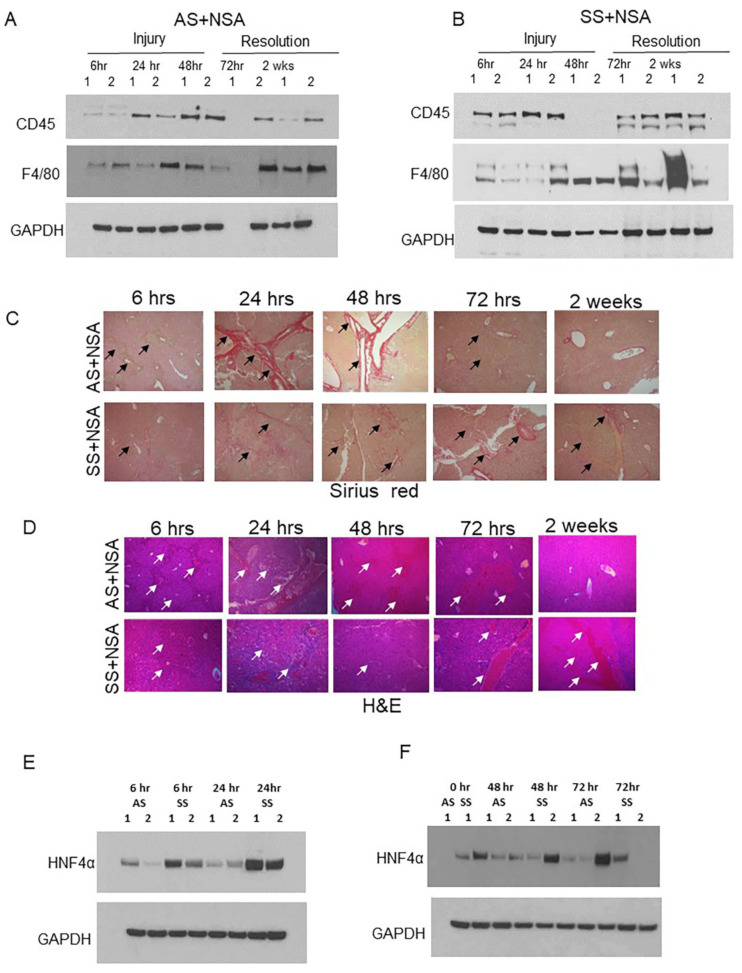
Kupffer cell activation is associated with reduced liver damage post-NSA treatment. (**A**,**B**) Representative western blot images showing expression of monocyte marker CD45 and Kupffer cells F4/80 in AS and SS mouse liver during the injury initiation (6 h, 24 h, and 48 h) and injury resolution phase (72 h and 2 weeks) post-NSA administration. (**C**,**D**) Representative IHC images showing the expression of H&E and Sirius red in AS and SS mouse liver during the injury initiation (6 h, 24 h, and 48 h) and injury resolution phase (72 h and 2 weeks) post-NSA administration. Image magnification: 60×. Arrows indicate vascular damage. (**E**,**F**) Representative western blot images showing expression of hepatocyte marker hepatocyte nuclear factor α (HNF4α) in AS and SS mouse liver during the injury initiation stage (6 h, 24 h, and 48 h) and injury resolution (72 h) post-NSA administration.

As we see a positive correlation between hepatic Kupffer cell activation and reduced liver damage, we hypothesized that hepatic Kupffer cells as well as monocyte-derived macrophages protect against NSA-induced liver damage by releasing different cytoprotectors. To confirm our hypothesis, we first analyzed the expression of hepatic Kupffer cell marker F4/80 and monocyte marker CD45 by western blot analysis. Remarkably, AS and SCD mouse liver showed opposite expression patterns of F4/80. As shown in Figure 3A, in AS mice, at the initial stages of injury, F4/80 protein level was low, whereas at later stages of injury (resolution phase), F4/80 protein expression was high. In contrast, SS mice showed significant enhancement of F4/80 protein at baseline, which continued during the initial stages of injury; however, was comparatively reduced during the resolution phases. CD45, which is a marker of Kupffer cells as well as liver monocytes, also showed a similar trend (Figure 3A,B, quantified in Figure S4A). H&E and Sirius red staining further confirmed exacerbated liver damage and liver fibrosis in AS mice at the injury phase, which was resolved at the resolution phase (Figure 3C). However, SS mice showed the complete opposite trend. Whereas the injury phase showed less hepatovascular damage, the resolution phase showed delayed resolution post-NSA administration (Figure 3C, zoomed in Figure S3A,B). As NSAs are known to cause significant necrosis of the hepatocytes [34], we next analyzed the expression of the hepatocyte-specific marker hepatocyte nuclear factor α (HNF4α) in AS and SCD/SS mice liver post-NSA treatment. As shown in Figure 3E (quantified in Figure S4C), hepatocyte death was more prominent in control mice as seen by the loss of HNF4α expression. SS mice liver did not show significant loss of HNF4α at 6 h, 24 h, 48 h, and 72 h of NSA treatment. Altogether, our data suggests that SS mice show activation of Kupffer cells at the injury stages of NSA treatment, which positively correlates with reduced liver fibrosis and hepatocyte death compared to control mouse liver. It also suggests that the delayed onset of injury in SS mice liver post-NSA treatment is associated with hepatic Kupffer cell activation.

### 3.4. Hepatic Kupffer Cell Promotes HO-1 Synthesis Which Induces Expression of Cytoprotective Enzyme Cyp3A11 in SS Mouse Liver

**Figure 4:**
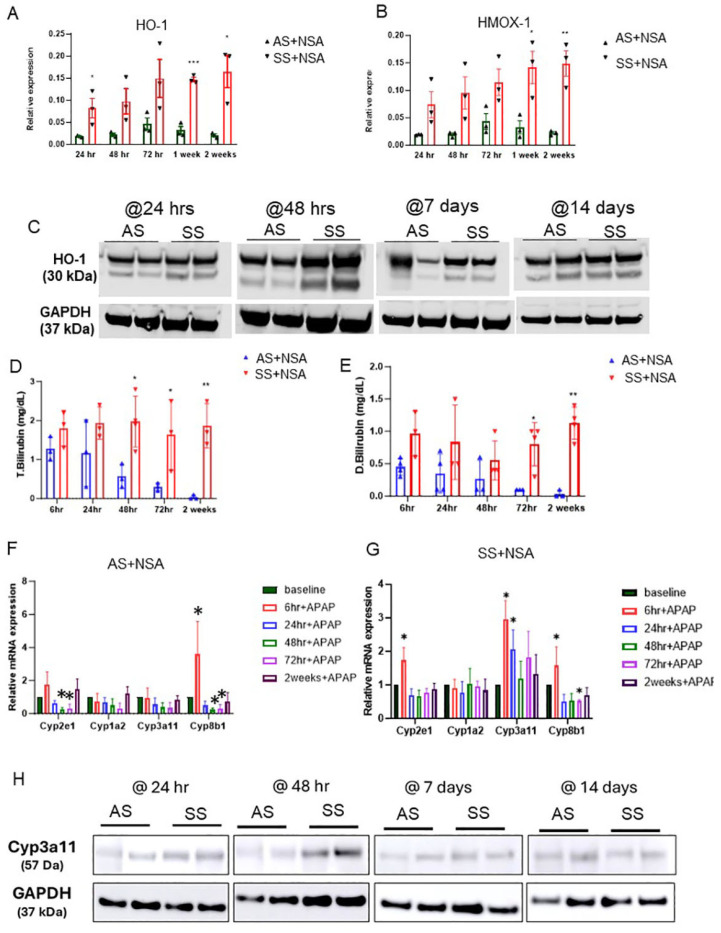
HO-1-mediated activation of Cyp3A11 protects against NSA-induced liver damage. (**A**,**B**) qRT-PCR analysis of HO-1 and HMOX1 in AS and SS mouse liver during the injury initiation (24 h and 48 h) and injury resolution phase (72 h, 1 week, and 2 weeks) post-NSA administration. (**C**) Representative western blot images showing the expression of HO-1 in AS and SS mouse liver during the injury initiation (24 h and 48 h) and injury resolution phase (1 week and 2 weeks) post-NSA administration. (**D**,**E**) qRT-PCR analyses showing expression of total bilirubin in AS and SS mouse liver during the injury initiation (6 h, 24 h, and 48 h) and injury resolution phase (72 h and 2 weeks) post-NSA administration. (**F**,**G**) qRT-PCR analyses showing expression of drug-metabolizing liver enzymes, including Cyp7A1 (cholesterol 7 alpha-hydroxylase), Cyp8B1 (cytochrome P450 family 8 subfamily B member 1), Cyp3A11 (cytochrome P450 3a11), and Cyp8b1 (cytochrome P450 family 8 subfamily B member 1) in AS and SS mouse liver during the injury initiation (6 h, 24 h, and 48 h) and injury resolution phase (72 h and 2 weeks) post-NSA administration. (**H**) Representative western blot images showing expression of Cyp3A11 in AS and SS mouse liver during the injury initiation (24 h and 48 h) and injury resolution phase (1 week and 2 weeks) post-NSA administration. *p*-value of significance * < 0.5. ** < 0.1. *** < 0.01.

To understand the mechanism of Kupffer cell-induced cytoprotective role in NSA-driven liver damage, we focused on HO-1, which is known to reduce oxidative stress and tissue damage in different disease contexts [15,16,17,18]. HO-1 is significantly upregulated in SCD to induce heme metabolism, and loss of HO-1 is shown to cause heme-iron accumulation, tissue damage, oxidative stress, and organ dysfunction [18,19,20]. Remarkably, when examined, we found two completely different patterns of HO-1 expression in control and SS mouse livers. In SS mouse liver, HO-1 synthesis (as analyzed by qRT-PCR) and protein expression (as analyzed by western blot) were significantly elevated at initial time points of NSA treatment (Figure 4A–C and Figure S4D). However, AS mice showed a unique expression pattern of HO-1, which negatively correlated with exacerbated liver damage. As seen in Figure 4A–C, HO-1 levels were reduced at the injury phase and peaked at 72 h (which is described as the peak time point of onset of injury resolution) post-NSA treatment. The synthesis of HO-1, as measured by the expression of the gene Heme oxygenase 1 (HMOX-1), also showed a similar expression pattern. 

To demonstrate enhanced HO-1 expression and activation of HO-1 signaling in SS mouse liver, we measured serum bilirubin level, a byproduct of HO-1-mediated heme degradation [15,16,17]. As shown in Figure 4D,E, total and direct bilirubin was significantly high at all different time points in SS mice. However, AS mice showed a decrease in bilirubin during the injury stage and a slight increase during the resolution phase suggestive of inactivation of HO-1 during acute injury please post-NSA administration. Total and direct bilirubin levels were highest at 72 h, which corresponded to enhanced HO-1 expression at the same time points in AS mouse liver (Figure 4D,E).

To further understand the HO-1-driven cytoprotection against NSA-induced hepatovascular damage, we next examined the known regulators of drug-metabolizing enzymes in the liver in AS and SS mice post-NSA treatment. Remarkably, as shown in Figure 4E,F, the drug-metabolizing/detoxifying enzyme Cyp3A11 showed a significant upregulation in SS mouse liver at baseline and injury stage and was reduced at the resolution phase (Figure 4F). However, AS mice showed a significant reduction of Cyp3A11 at initial time points of injury, whereas at the resolution phase, the levels of Cyp3A11 were elevated, mimicking the HO-1 expression pattern (Figure 4G and Figure S4D) in these mice post-NSA treatment.

**Figure 5:**
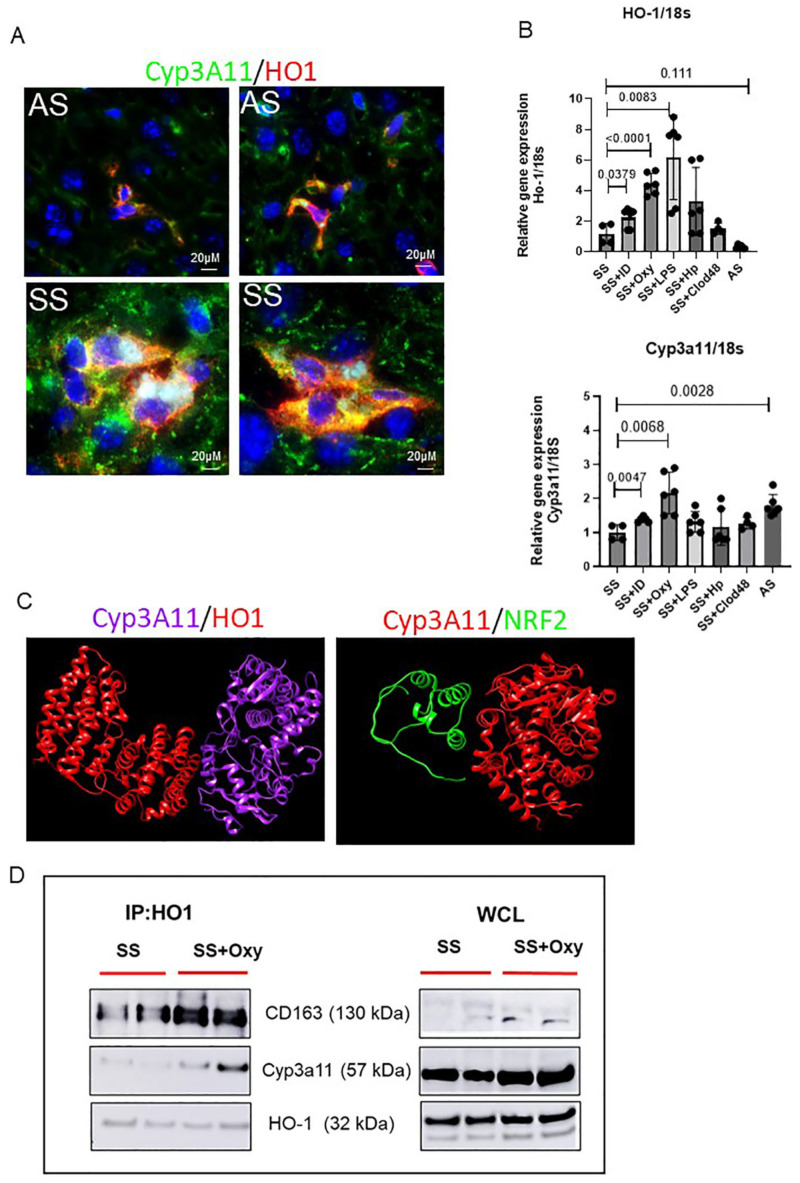
Characterization of HO-1–Cyp3A11 interaction in AS and SS mouse liver. (**A**) Representative immunofluorescence images of liver tissue section showing the colocalization of HO-1 and Cyp3A11 in AS and SS mouse liver. (**B**) qRT-PCR analysis of HO-1 and Cyp3A11 in AS and SS mouse liver at baseline and in SS mouse liver post-treatment with iron dextran, oxyhemoglobin, LPS, and clodronate liposomes. (**C**) Structural biology prediction of Cyp3A11 with HO-1 and NRF2 binding. (**D**) Coimmunoprecipitation analysis of HO-1 with Cyp3A11 and cd163 in SS mouse liver at baseline and post-oxyHb treatment.

We hypothesized that Cyp3A11 levels are positively regulated by HO-1. To prove that we first performed an immunofluorescence assay of HO-1 and Cyp3A11 in AS and SS mouse liver. As shown in Figure 5A, both AS and SS mice showed colocalization of Cyp3A11 and HO-1, which was strongly increased in SS mice liver. Next, we examined how HO-1 and Cyp3A11 levels are regulated in SS mice liver. As shown in Figure 5B, qRT-PCR analysis confirmed that, whereas any form of acute injury (oxy-Hb treatment, LPS, iron dextran treatment, and haptoglobin administration) increased both HO-1 and Cyp3A11 levels, clodronate-mediated macrophage depletion reduced HO-1 and Cyp3A11 levels significantly. This finding further confirms a macrophage-specific activation of these two proteins.

Finally, to examine the possibility of an interaction between HO-1 and Cyp3A11, we used two different complementary approaches. First, to understand the interaction of HO-1 and Cyp3A11, we performed a bioinformatic analysis. The crystal structures of both HO-1 and Cyp3A11 were retrieved from the Protein Data Bank (www.rcsb.org accessed on 11 August 2024) and UniProt. The potential interactions of Cyp3a11 and HO1 were carried out with the pyDockWEB platform (https://life.bsc.es/servlet/pydock accessed on 11 August 2024), followed by visualization of the docked complex using the UCSF Chimera molecular visualization program. As shown in Figure 5C, the algorithm predicted a very strong binding affinity between Cyp3A11 and HO-1. However, when examined, we did not find a strong binding affinity between NRF2 and Cyp3A11, suggestive of an NRF2-independent binding of HO-1 and Cyp3A11. Second, to confirm the interaction between Cyp3A11 and HO-1, we performed a coimmunoprecipitation assay using the mouse liver tissue. HO-1 and Cyp3A11 formed a complex, as shown in Figure 5D, which was stronger in oxy-Hb-treated SS mice liver (Figure 5D). Taken together, our data suggested that activation of Kupffer cells is strongly associated with increased synthesis of both HO-1 and Cyp3A11. It also suggests that activated Kupffer cells release HO-1, which induces the synthesis of Cyp3A11, promoting cytoprotection against oxidative damage and stress.

## 4. Discussion

Acute pain crisis is one of the leading causes of hospitalization in SCD patients [1]. Both steroidal and non-steroidal analgesics (NSA) are commonly utilized to treat SCD-related pain crises [38,39]. Previous studies have demonstrated side effects and altered clearance of opioids in SCD patients [40]. However, the effect of non-steroidal analgesics (NSAs) on the SS mouse liver was not previously examined. Identifying the mechanisms of hepatic metabolism of analgesics using animal models is critical for mitigating the long-term adverse effects of drug metabolites. In this study, we sought to identify the effects of NSAs on the liver of SS mice using real-time intravital imaging of the liver as well as mechanistic approaches. Remarkably, we found that SS mice were better able to resist NSA-associated acute insult compared to control mice. However, injury resolution and wound healing were delayed in SS mice, causing persistent mild liver damage. Mechanistically, we found that delayed onset of injury was associated with an activated Kupffer cell population in SS mouse liver. Activated Kupffer cells released HO-1, which induced the synthesis of Cyp3A11 in SS mice at baseline to protect against NSA-associated liver damage. Thus, the baseline presence of heme-associated cytoprotectors can mitigate many of the harmful effects of NSA byproducts.

Previous studies have demonstrated that higher HO-1 expression is associated with improved vasoocclusive crisis and inhibition of vascular inflammation in SCD [18,19,20]. The enzymatic byproducts of HO-1, including carbon monoxide, have been demonstrated to improve the degree of erythrocyte sickling in SCD patients [21,22,23]. Preclinical studies have also confirmed the presence of elevated levels of HO1 in patrolling monocytes, which protects against vasoocclusive pain crisis [27,28,29]. However, a direct link between HO-1 and pain analgesics (NSA) used in SCD was not previously documented. In this study, we found that the severity of the liver damage is inversely proportional to the amount of HO-1 in both SS and control mice. We also show that HO-1 levels show a positive correlation with Cyp3A11 expression, and an increase in hemoglobin/heme or oxidative stress causes an enhancement of both these enzymes. Similarly, clodronate liposome-mediated Kupffer cell depletion inhibited the synthesis of both HO-1 and Cyp3A11. Finally, we found a strong positive binding affinity, as evident from both structural biology and biochemical analyses, confirming a strong influence of HO-1 on Cyp3A11.

Cyp3A11 is a member of the cytochrome P450 regulatory enzyme family found in the liver and has a role in hepatic drug metabolism [41,42]. Cyp3A11 also exhibits high expression in human cell lines that express NADPH-cytochrome P450 reductase [43]. Various cytotoxic conditions, including α-toxin B1, benzoflavone, and other xenobiotics, are known to induce Cyp3A11 expression in the liver [43]. Apart from xenobiotics, prior studies have also confirmed several positive and negative regulators of Cyp3A11, including pregnane X receptor (PXR), transcription factor E4bp4, toll-like receptor 4 (TLR4), and the chromatin remodeling protein BRG1 [41,42,43]. However, HO-1 was not previously reported to regulate Cyp3A11 level. When examined, we found enhanced expression of both Cyp3A11 and HO-1 following iron or oxy-Hb treatment (Figure S5A–D). Future studies using knockout models can be useful in understanding the direct association between these two proteins. It would also be useful to examine if Cyp3A11 has any potential direct role in heme metabolism in SCD, as well as other hemolytic disease conditions. We found elevated levels of Cyp3A11 in SS mice. However, SCD patients were not previously associated with elevated levels of Cyp3A11. Thus, it would be beneficial to examine the human relevance of these findings. Both HO-1 and Cyp3A11 are not directly associated with any current SCD clinical trials, and our results also highlight the potential novel therapeutic avenues of these two compounds in non-steroidal analgesic-driven end-organ damage, as well as hemolytic complications associated with SCD.

**Figure 6:**
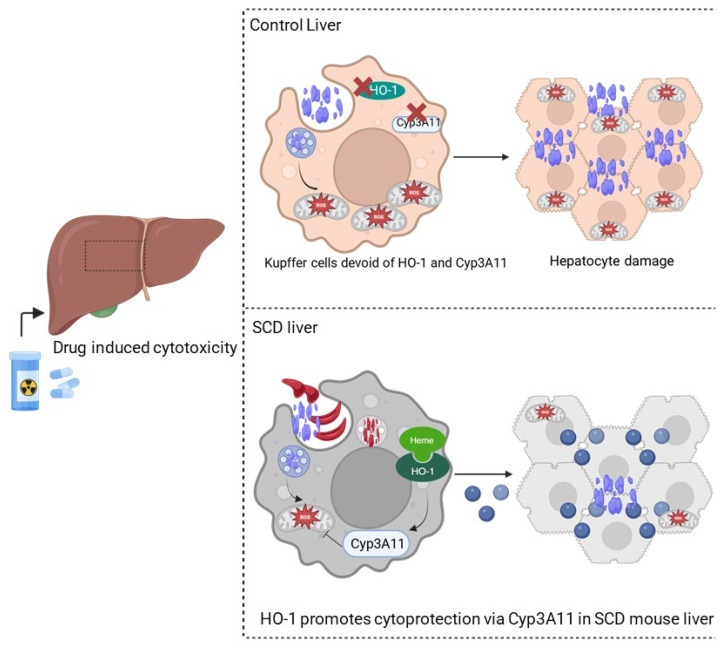
HO-1-mediated activation of Cyp3A11 protects against non-steroidal analgesic (NSA)-associated liver damage. Schematic showing NSA-induced liver damage in control and SS mouse livers. In control mouse liver, due to the absence of HO-1 and Cyp3A11, there is no protection against hepatocyte damage. However, in SS mouse liver, the presence of HO-1 and Cyp3A11 protects against oxidative stress and hepatocyte death and reduces the adverse effects of NSA on the liver.

Based on our findings, we propose the following model (Figure 6): in control mouse liver, in the absence of severe hemolysis, HO-1 levels are maintained low at baseline. However, in SCD and other hemolytic disease conditions, due to persistent hemolysis, heme is accumulated in organs, causing significant upregulation of HO-1, which acts as an inducer of Cyp3A11, and HO-1 and Cyp3A11 together inhibit tissue accumulation of xenobiotics by potentially inducing the NADPH driven antioxidative stress mechanism. Although heme is considered a damage-associated molecular pattern (DAMP) that causes inflammation and tissue injury, this study might highlight the presence of a heme-mediated potential feedback loop existing as an adaptive defense mechanism in the body via HO-1–Cyp3A11 nexus to combat DAMP and other xenobiotics-associated organ damage.

Our study does have a few limitations that need to be addressed in the future. For example, the role of HO-1 in Cyp3A11 synthesis should be confirmed in other organs involved in drug metabolism and clearance including the kidney. It would be also interesting to decipher if opioid metabolism is also positively regulated by the HO-1–Cyp3A11 complex. Finally, the signaling pathway downstream of HO-1 and Cyp3A11 signaling should be identified for novel therapeutic benefits.

In conclusion, utilizing real-time intravital imaging of the Townes SS mouse model, we identified the novel roles of both HO-1 and Cyp3A11 as potential cytoprotectors of NSA-driven acute organ damage. These preclinical results show robust proof-of-concept for HO-1 and Cyp3A11 against NSA-induced liver damage in the Townes model of SCD, paving the way for further development of these compounds as possible new treatments for end-organ damage in SCD.

## Data Availability

The original contributions presented in this study are included in the article/Supplementary Materials. Further inquiries can be directed to the corresponding author.

## Supplementary Materials

The following supporting information can be downloaded at https://www.mdpi.com/article/10.3390/cells14030194/s1. Figure S1: Immunohistochemical characterization of early phases of non-steroidal liver damage in AS and SS mice. Figure S2: Immunohistochemical characterization of early phase and resolution phases of non-steroidal liver damage in AS and SS mice. Figure S3: Immunohistochemical characterization of late phase of non-steroidal liver damage in AS and SS mice. Figure S4: Comparison of protein expression in AS and SS mouse liver post NSA administration. Figure S5: Regulation of HO-1/Cyp3A11 by iron heme signalling. Table S1: Primary Antibodies used for Western Blot analysis. Table S2: Primary Antibodies used for IHC/IF analysis. Table S3: Sequences of primers used in this study.
