# Supplementary material for: Heme-Oxygenase 1 Mediated Activation of Cyp3A11 Protects Against Non-Steroidal Pain Analgesics Induced Acute Liver Damage in Sickle Cell Disease Mice
Source: Cells. 2025 Jan 28;14(3):194. doi: 10.3390/cells14030194 (PMC11817884; doi:10.3390/cells14030194)
Supplement: Supplementary file 1 [file cells-14-00194-s001.zip › cells-3408057-supplementary.pdf]

Supplemental Material for

Heme-oxygenase 1 mediated activation of Cyp3a11 protects against non-steroidal analgesics induced acute liver damage in Sickle Cell Disease mice.

Ravi Vats[1,2,], Ramakrishna Ungalara[1,2,], Rikesh Dubey[1,2], Prithu Sundd[1,2] and Tirthadipa Pradhan-Sundd[1,2,*].

1. Versiti Blood Research Institute, Wisconsin, USA
2. Medical College of Wisconsin, Wisconsin, USA

*To whom correspondence should be addressed:

Tirthadipa Pradhan-Sundd, PhD

Transfusion medicine vascular biology and cell therapy

Versiti Blood Research Institute and Blood Center of Wisconsin

8733 West Watertown Plank Road,

Milwaukee, WI

Email: tpradhan@versiti.org

Table of contents

Supplemental Methods

Supplemental Figures and legends

Supplemental Videos and legends

Supplemental Methods

Supplemental Table S1: Primary Antibodies used for Western Blot analysis.

Western Blot	Concentration used	Company	Catalogue / Lot No:	Original concentration
GAPDH	0.042 µg/mL	Cell Signaling Technology	2118L	42 µg/mL
Clec4F	0.025 µg/mL	R&D Systems	AF2784	25 µg/mL
F4/80 (D2S9R) XP	0.435 µg/ml	Cell Signaling Technology,	70076S	435 µg/mL
HO-1	0.5 mg/ml	Abcam	AB189491	500 µg/mL
Cyp3a11	0.1 µg/ml	Santacruz	SC53246	100 µg/ml

Supplemental Table S2: Primary Antibodies used for IHC/IF analysis.

Name	Concentration	Company	Catalogue / Lot No:	Original concentration
CD163	1:100 dilution	Bioss	Bs-2527R<br>Lot #: BB06141205	1µg/µL
CLEC4F	1:100 dilution	R&D Systems	AF2784<br>Lot #: VLB0321061	0.2 mg/mL
CD11b	1:100 dilution	Novus Biologicals	NB110-89474SS<br>Lot #: D109642	1 mg/mL
F4/80 (tagged AF 647)	2.5 - 10 µg/mL	Bio legend	123121<br>Lot #: B33665	0.5 mg/mL
HO-1	1:250 dilution	Abcam	ab189491<br>Lot #: GR3348210-13	0.501 mg/ml

Supplemental Table S3: Sequences of primers used in this study

Gene name	Forward Primer	Reverse Primer
HO-1	5’-TCCCAGACACCGCTCCTCCAG-3’	5’-GGATTTGGGGCTGCTGGTTTC-3’
Cyp3A11	5'-TTCTGTCTTCACAAACCGGC -3'	5'-GGGGGACAGCAAAGCTCTAT -3'
GAPDH	5’TGACCTCAACTACATGGTCTACA-3’	R: 5’-CTTCCCATTCTCGGCCTTG-3’
Col1a1,	CATGTTCAGCTTTGTGGACCT	GCAGCTGACTTCAGGGATGT
Col1a2	TAAGGGTACCGCTGGAGAAC	CTCCCTGAGCTCCAGCTTCT
Col3a1	AGAGGACCACGTGGACAAAG	TGAGCAGCAAAGTTCCCAGT
Tgfbeta.	GTGTGGAGCAACATGTGGAACTCTA	TTGGTTCAGCCACTGCCGTA
Alpha SMA	GTTCAGTGGTGCCTCTGTCA	ACTGGGACGACAGGAAAAG
Cyp2e1	CGCATGGAACTGTTTCTGC	CAATTGTAACAGGGCTGAGGTC
Cyp1a2	CAAGAGGTTTAAGACCTTCAATGATAAC	AAAGATGTCATTGACAATGTTGACAAT
Cyp8b1	GCCTTCAAGTATGATCGGTTCCT	GATCTTCTTGCCCGACTTGTAGA

Supplemental Figures and legends

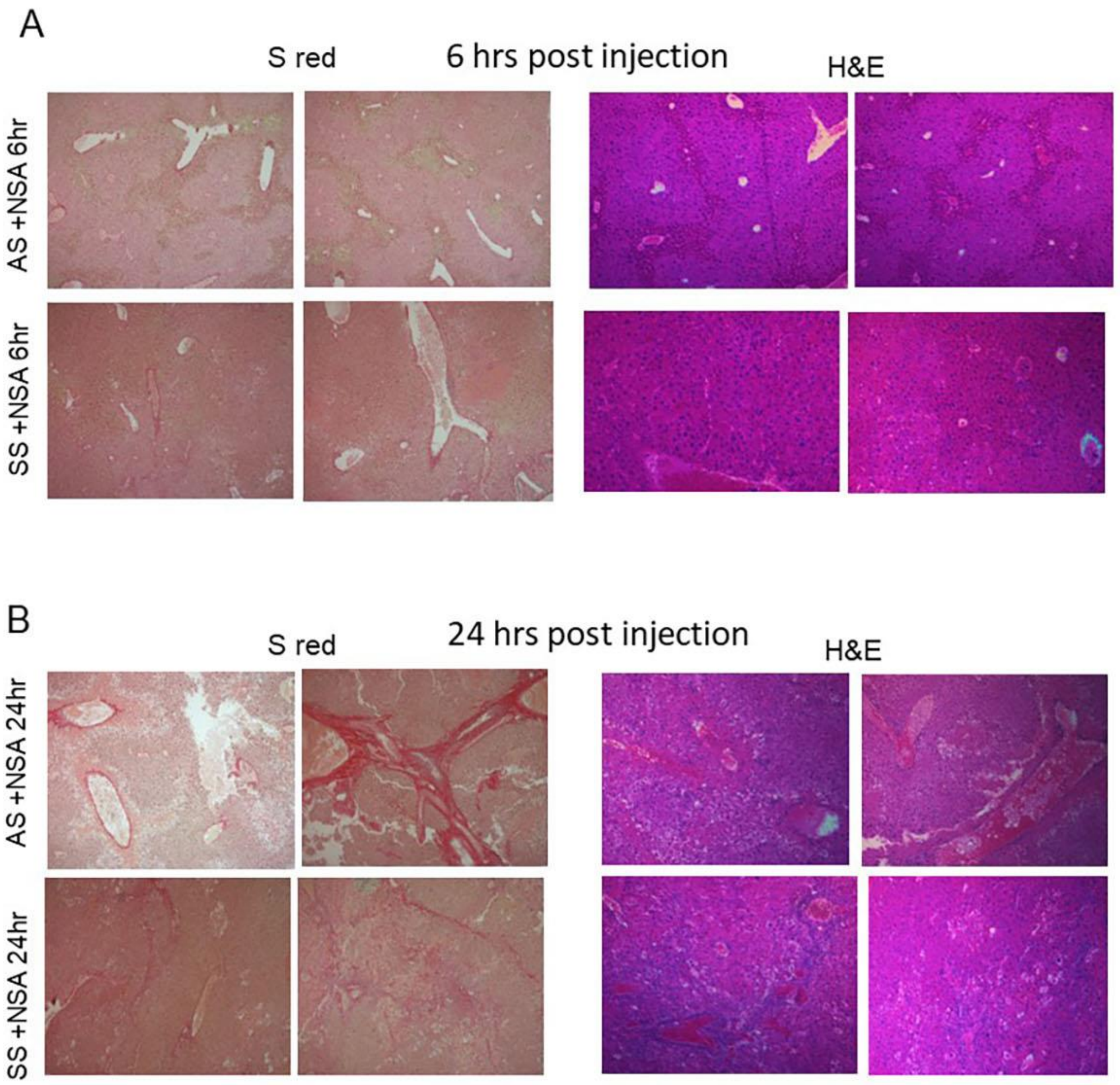

Figure S1: Immunohistochemical characterization of early phases of non-steroidal liver damage in AS and SS mice. **(A-B)** Representative IHC images of H&E and Sirius Red staining showing exacerbated liver damage, vascular necrosis, and fibrosis in AS mice. However, SS mice showed less hepatovascular damage at 6 as well as 24 hrs. post NSA administration.

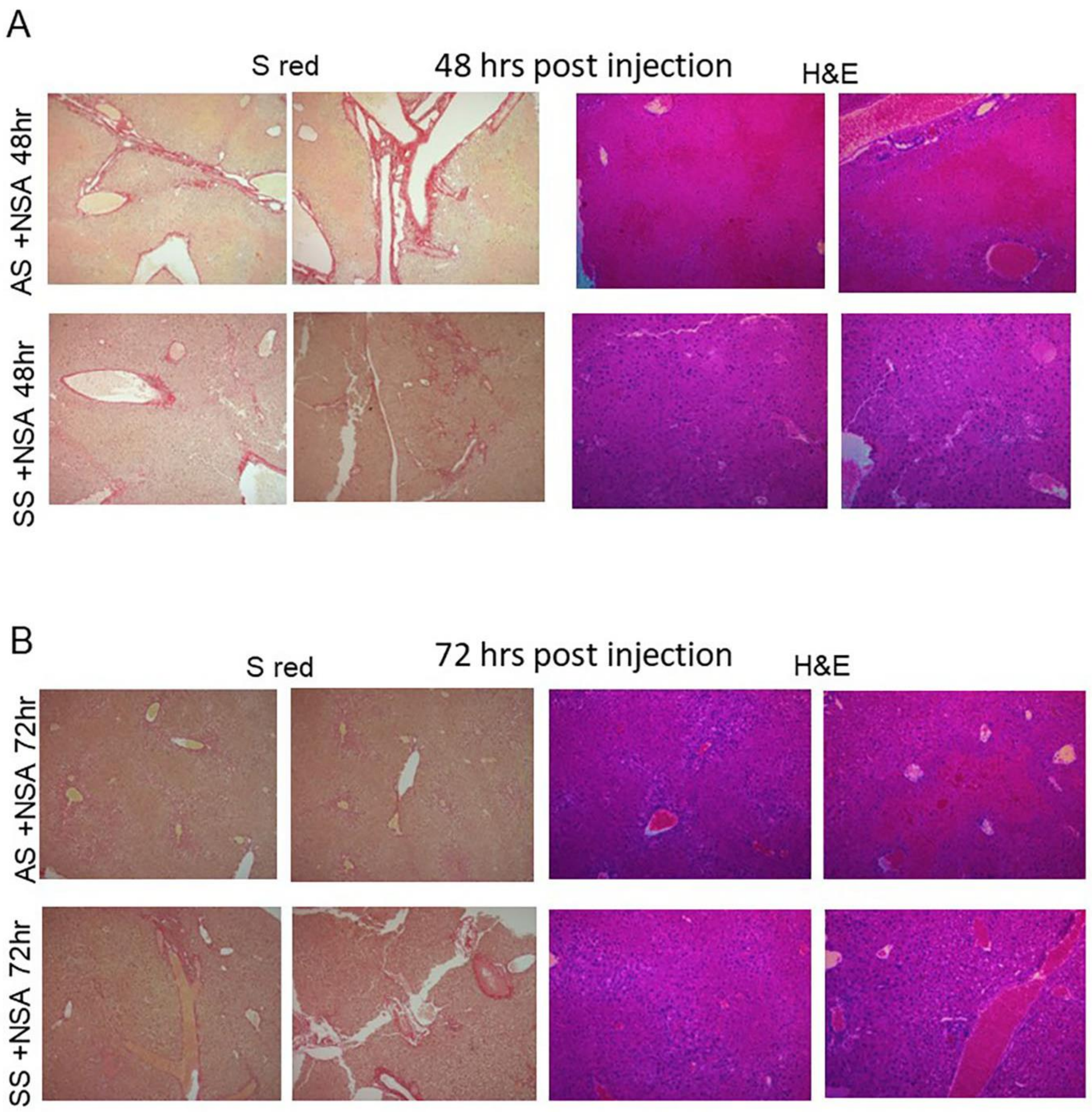

Figure S2: Immunohistochemical characterization of early phase and resolution phases of non-steroidal liver damage in AS and SS mice. **(A-B)** Representative IHC images of H&E and Sirius Red staining showing exacerbated liver damage, vascular necrosis and fibrosis in AS mice at 48 hrs. However, at 72 hrs. (resolution phase) AS mice show significant improvement of hepatovascular damage. However, SS mice showed less hepatovascular damage at 48 hrs. post NSA administration which got aggravated at 72 hrs. post NSA administration.

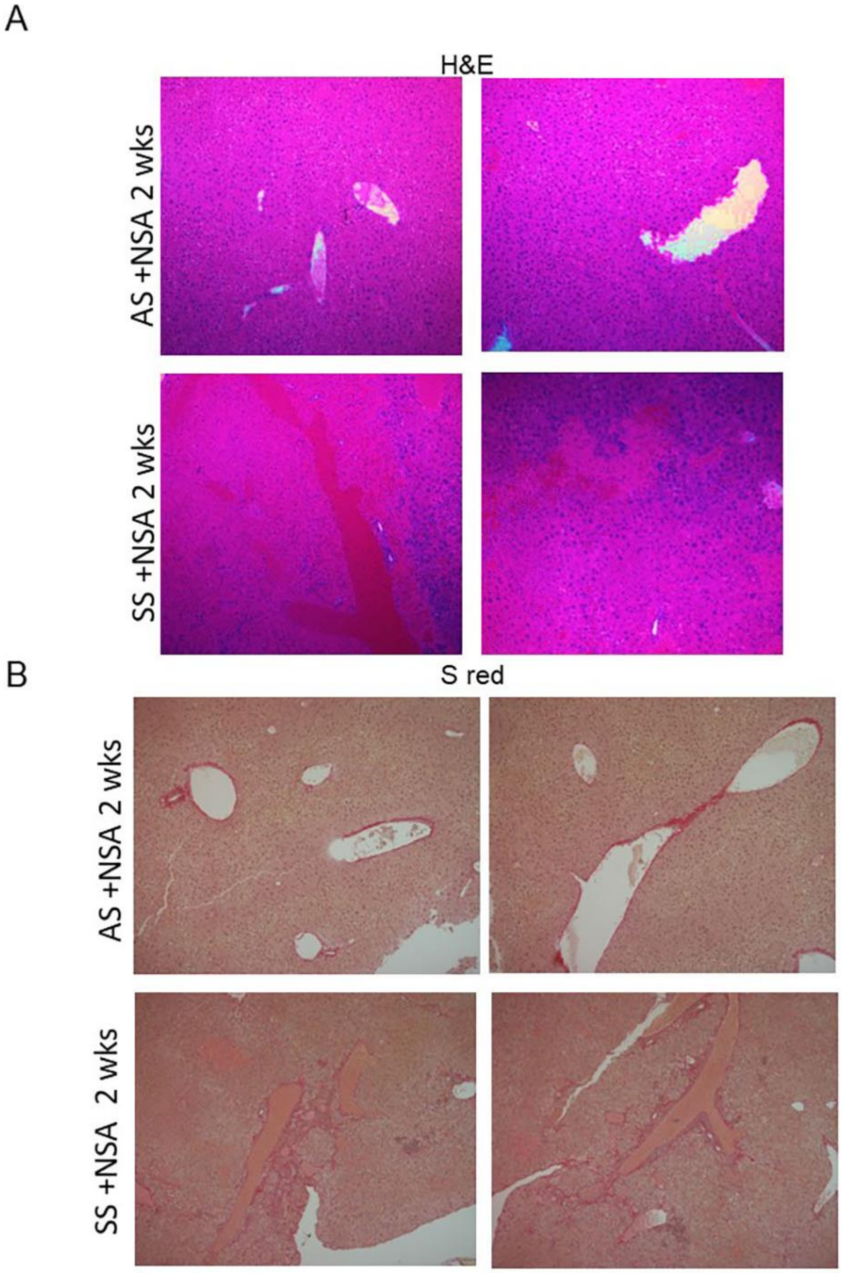

Figure S3: Immunohistochemical characterization of late phase of non-steroidal liver damage in AS and SS mice. **(A-B)** Representative IHC images of H&E and Sirius Red staining showing exacerbated liver damage, vascular necrosis, and fibrosis in SS mice at both 7 and 14 days post NSA administration which is completely resolved in AS mice at these timepoints.

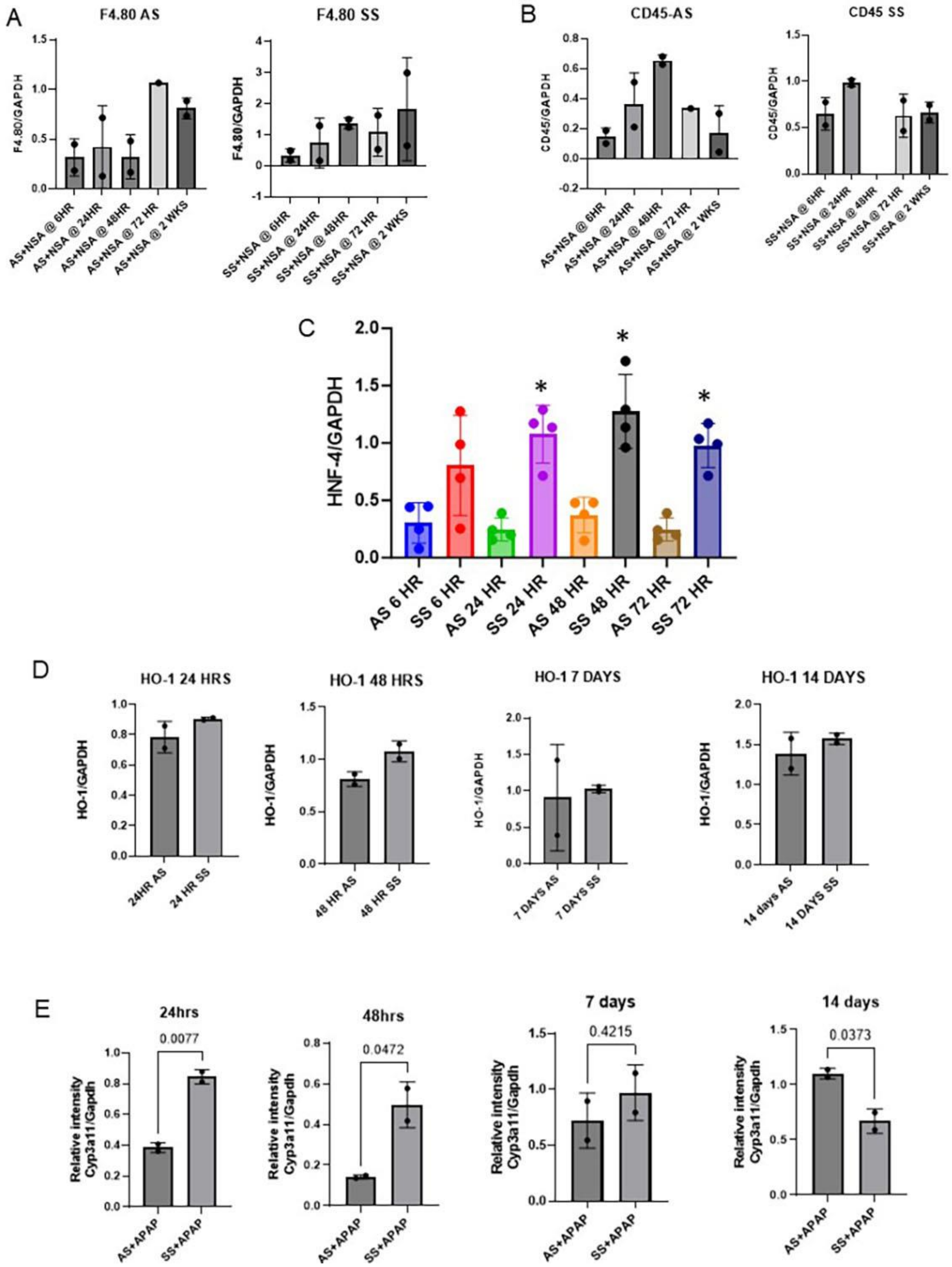

Figure S4: Comparison of protein expression in AS and SS mouse liver post NSA administration. **(A)** Densitometric analysis exhibiting the quantification of F4/80 expression in AS and SS mouse liver post NSA treatment. **(B)** Densitometric analysis exhibiting the quantification of CD45 expression in AS and SS mouse liver post NSA treatment. **(C)** Densitometric analysis exhibiting the quantification of HNF4α expression in AS and SS mouse liver post NSA treatment. **(D)** Densitometric analysis exhibiting the quantification of HO-1 expression in AS and SS mouse liver post NSA treatment. **(E)** Densitometric analysis exhibiting the quantification

of Cyp3A11 expression in AS and SS mouse liver post NSA treatment. Each data point was represented as mean ±SEM.

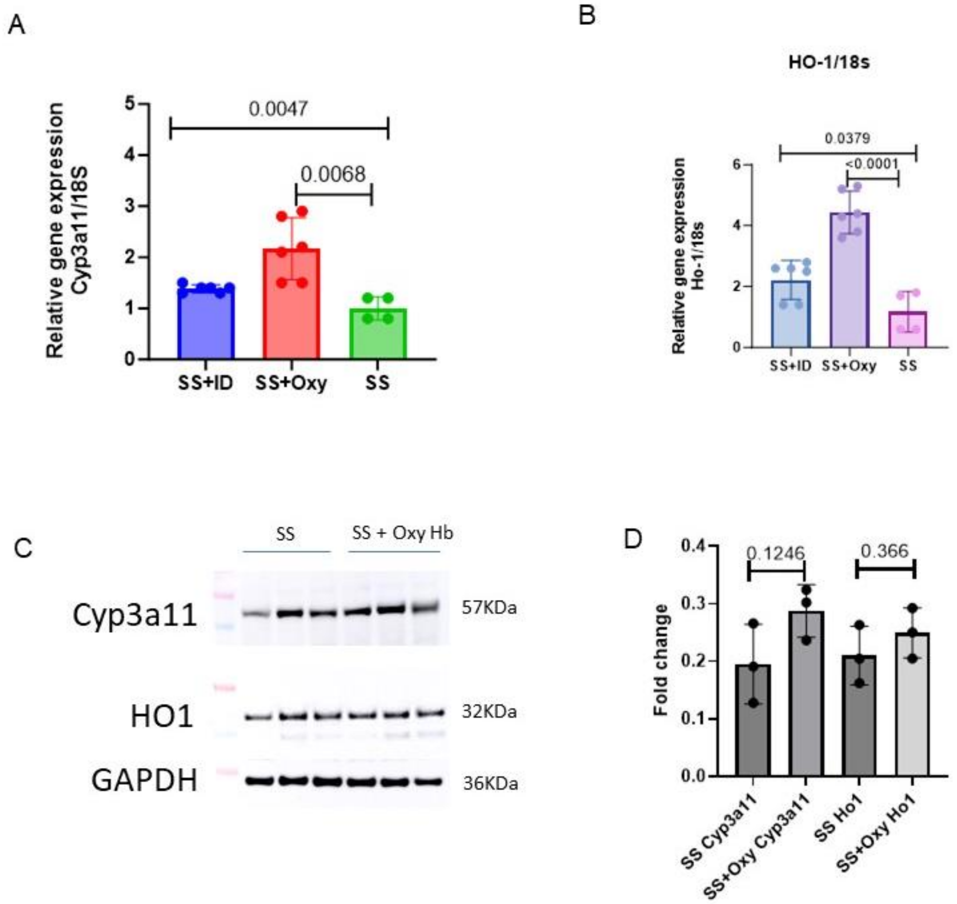

Figure S5: Regulation of HO-1/Cyp3A11 by iron heme signalling. **(A)** Quantitative RT-PCR data showing the effect on Cyp3A11 expression post iron dextran and oxy-Hb treatment. **(B)** Quantitative RT-PCR data showing the effect on HO-1 expression post iron dextran and oxy-Hb treatment. **(C)** Representative western blot analysis showing the expression of cyp3a11 and HO1 in SS mouse liver at baseline and post oxy-Hemoglobin treatment. **(D)** Densitometric analysis exhibiting the quantification of Cyp3A11 and HO1 expression in SS mouse liver post oxy Hb treatment. Each data point was represented as mean ±SEM.

Supplemental Movies:

Movie S1-2. Visualization of blood flow in a control (AS) mouse 24 hrs post NSA treatment after administration of TXR dextran and F4/80 prior to imaging. The sinusoids in AS mouse liver visualized by carotid artery injection of TXR-dextran (red) and AF-F4/80 was used to visualize the Kupffer cells. Scale bar 20 uM.

Movie S3-4. Visualization of blood flow in a SCD (SS) mouse 24 hrs post NSA treatment after administration of TXR dextran and F4/80 prior to imaging. The sinusoids in SCD mouse liver visualized by carotid artery injection of TXR-dextran (red) and AF-F4/80 was used to visualize the Kupffer cells. Scale bar 20 uM.

Movie S5-6. Visualization of blood flow in a control (AS) mouse 7 days post NSA treatment after administration of TXR dextran and F4/80 prior to imaging. The sinusoids in AS mouse liver visualized by carotid artery injection of TXR-dextran (red) and AF-F4/80 was used to visualize the Kupffer cells. Scale bar 20 uM.

Movie S-8. Visualization of blood flow in a SCD (SS) mouse 7 days post NSA treatment after administration of TXR dextran and F4/80 prior to imaging. The sinusoids in SCD mouse liver visualized by carotid artery injection of TXR-dextran (red) and AF-F4/80 was used to visualize the Kupffer cells. Scale bar 20 uM.

Movie S9-10. Visualization of blood flow in a control (AS) mouse 14 days post NSA treatment after administration of TXR dextran and F4/80 prior to imaging. The sinusoids in AS mouse liver visualized by carotid artery injection of TXR-dextran (red) and AF-F4/80 was used to visualize the Kupffer cells. Scale bar 20 uM.

Movie S11-12. Visualization of blood flow in a SCD (SS) mouse 14 days post NSA treatment after administration of TXR dextran and F4/80 prior to imaging. The sinusoids in SCD mouse liver visualized by carotid artery injection of TXR-dextran (red) and AF-F4/80 was used to visualize the Kupffer cells. Scale bar 20 uM.
